# Supplementary material for: Perceptual expertise with Chinese characters predicts Chinese reading performance among Hong Kong Chinese children with developmental dyslexia
Source: PLoS One. 2021 Jan 22;16(1):e0243440. doi: 10.1371/journal.pone.0243440 (PMC7822259; doi:10.1371/journal.pone.0243440)
Supplement: Attachment — Submitted filename: ResponseLetterToReviewers_v4.pdf [file pone.0243440.s002.pdf]

Dear Professor Protopapas,

Thank you for your action letter. We are very grateful for getting the constructive feedback and references from the reviewers, from which we learned a lot ourselves. We believe that the revisions strengthened the manuscript and that our findings are now better communicated to researchers from different backgrounds.

We have now responded to the comments below and revised the manuscript accordingly. We hope you will find the manuscript suitable for publication.

Thank you again for your attention.

Yetta Wong & Alan Wong

****

PONE-D-20-07055R1
Perceptual expertise with Chinese characters predicts Chinese reading performance among Hong Kong Chinese children with developmental dyslexia
PLOS ONE

Dear Dr. Wong,

Thank you for submitting your manuscript to PLOS ONE. After careful consideration, we feel that it has merit but does not fully meet PLOS ONE's publication criteria as it currently stands. Therefore, we invite you to submit a revised version of the manuscript that addresses the points raised during the review process.

In particular, the reviewers are unanimous in their overall positive evaluation, as well as in their evaluation that your manuscript only partially fulfills the essential criterion of being technically sound with conclusions fully supported by the data, and they provide several constructive suggestions for improvement on this front. Although there are several points raised, and many additional sources of information recommended to be taken into account, it seems possible to me that a revised manuscript may be able to address these criticisms, and I would therefore like to give you an opportunity to do that in a revision.

Please submit your revised manuscript by Aug 20 2020 11:59PM. If you will need more time than this to complete your revisions, please reply to this message or contact the journal office at plosone@plos.org. When you're ready to submit your revision, log on to https://www.editorialmanager.com/pone/ and select the 'Submissions Needing Revision' folder to locate your manuscript file.

Please include the following items when submitting your revised manuscript:

- A rebuttal letter that responds to each point raised by the academic editor and reviewer(s). You should upload this letter as a separate file labeled 'Response to Reviewers'.
- A marked-up copy of your manuscript that highlights changes made to the original version. You should upload this as a separate file labeled 'Revised Manuscript with Track Changes'.
- An unmarked version of your revised paper without tracked changes. You should upload this as a separate file labeled 'Manuscript'.

If you would like to make changes to your financial disclosure, please include your updated statement in your cover letter. Guidelines for resubmitting your figure files are available below the reviewer comments at the end of this letter.

If applicable, we recommend that you deposit your laboratory protocols in protocols.io to enhance the reproducibility of your results. Protocols.io assigns your protocol its own identifier (DOI) so that it can be cited independently in the future. For instructions see: http://journals.plos.org/plosone/s/submission-guidelines#loc-laboratory-protocols

We look forward to receiving your revised manuscript.

Kind regards,

Athanassios Protopapas
Academic Editor
PLOS ONE

[Note: HTML markup is below. Please do not edit.]

Reviewers' comments:

Reviewer's Responses to Questions

Comments to the Author

1. If the authors have adequately addressed your comments raised in a previous round of review and you feel that this manuscript is now acceptable for publication, you may indicate that here to bypass the “Comments to the Author” section, enter your conflict of interest statement in the “Confidential to Editor” section, and submit your "Accept" recommendation.

Reviewer #1: (No Response)

Reviewer #2: All comments have been addressed

Reviewer #3: (No Response)

---

2. Is the manuscript technically sound, and do the data support the conclusions?

The manuscript must describe a technically sound piece of scientific research with data that supports the conclusions. Experiments must have been conducted rigorously, with appropriate controls, replication, and sample sizes. The conclusions must be drawn appropriately based on the data presented.

Reviewer #1: Partly

Reviewer #2: Partly

Reviewer #3: Partly

---

3. Has the statistical analysis been performed appropriately and rigorously?

Reviewer #1: Yes

Reviewer #2: Yes

Reviewer #3: Yes

---

4. Have the authors made all data underlying the findings in their manuscript fully available?

The PLOS Data policy requires authors to make all data underlying the findings described in their manuscript fully available without restriction, with rare exception (please refer to the Data Availability Statement in the manuscript PDF file). The data should be provided as part of the manuscript or its supporting information, or deposited to a public repository. For example, in addition to summary statistics, the data points behind means, medians and variance measures should be available. If there are restrictions on publicly

sharing data—e.g. participant privacy or use of data from a third party—those must be specified.

Reviewer #1: Yes

Reviewer #2: Yes

Reviewer #3: Yes

---

5. Is the manuscript presented in an intelligible fashion and written in standard English?

PLOS ONE does not copyedit accepted manuscripts, so the language in submitted articles must be clear, correct, and unambiguous. Any typographical or grammatical errors should be corrected at revision, so please note any specific errors here.

Reviewer #1: Yes

Reviewer #2: Yes

Reviewer #3: Yes

---

6. Review Comments to the Author

Please use the space provided to explain your answers to the questions above. You may also include additional comments for the author, including concerns about dual publication, research ethics, or publication ethics. (Please upload your review as an attachment if it exceeds 20,000 characters)

Reviewer #1: I appreciate the opportunity given or reviewing this paper which can potentially contribute to this society. However, there are a few concerns I have as below.
1. Literature review: The authors mentioned "children with developmental dyslexia fail to develop perceptual expertise with word stimuli." on p.5. However, this is quite an under debate. This issue is about the argument

whether dyslexic children have talents in their visuospatial abilities which have been discussed for a long time in either alphabetic languages (e.g., Brunswick, Martin, & Marzano, 2010) or Chinese (e.g., Wang & Yang, 2013). It's more about whether it focuses on detailed or gross information of the visual stimuli. So, the authors are suggested to make a more solid argument here with appropriate citations.

Brunswick, N., Martin, G. N., & Marzano, L. (2010). Visuospatial superiority in developmental dyslexia: Myth or reality?. Learning and Individual Differences, 20(5), 421-426.
Wang, L. C., & Yang, H. M. (2011). The comparison of the visuo-spatial abilities of dyslexic and normal students in Taiwan and Hong Kong. Research in Developmental Disabilities, 32(3), 1052-1057.

RESPONSE: Thank you for the suggestion and for pointing us to the references. We have now added a paragraph on p.8 to discuss how these two constructs are different, which is also included below:

> *Although words in measures of perceptual expertise are often located in different visuospatial locations horizontally (Figure 1), perceptual expertise with words does not simply refer to general visuospatial abilities, with which children with dyslexia are often suggested to be superior (82). In visuospatial tasks, participants are often required to report complex visual patterns from memory, perform mental depth rotation of three-dimensional objects, or reproduce a map after exploring a three-dimensional virtual environment (82,83). The task demands of these tasks heavily involve visual working memory and/or long-term memory, mental imagery of the structure of three-dimensional objects, and judgment of spatial distance and relationship between multiple objects. In contrast, perceptual expertise with words emphasizes on fine-level discrimination between two-dimensional word stimuli, which does not involve any spatial judgment or spatial imagination of the stimuli (e.g., performing plane- or depth-rotation with words, or judging the distance and positions between the words). With a brief time gap between stimulus presentation and report, the requirement on visual working memory is relatively minimal. Overall, perceptual expertise with words focus on abilities that are quite distinct from that captured by visuospatial tasks.*

2. Research questions: The two research questions are quite the same.

RESPONSE: We now clarify on p.10 that the 1st research question is about the correlation between perceptual expertise with words and reading, and the 2nd is about whether perceptual expertise with words is a unique predictor of reading above and beyond other measures.

3. Research questions: The authors mentioned "Ceiling effects could easily result if the tasks were performed by typically developing children. Hence comparing the role of perceptual expertise with words in normal readers and readers with dyslexia was out of the scope of this study." on p.9. However, the claimed ceiling effect should have proof, otherwise, it is not convincible. For the research in this field, it's not normal to find the study without a reference group, especially the targeted issue isn't a very popular and well-accpted one, as deficient orthographic knowledge, in Chinese contexts.

RESPONSE: Since our study aimed to examine individual differences *within* those with dyslexia, it is important for *all* tasks to be off ceiling and floor. This issue might not be as much of a concern for studies with other goals, e.g., that performed group comparison between children with dyslexia and typically developing children (e.g., Brunswick et al., 2010; Gabay et al., 2017) or that performed logistic regression to categorize children as those with dyslexia or not (e.g., Shu et al., 2006).

Indeed, ceiling effects are not uncommon even for studies conducted by researchers who have been testing children with dyslexia for years (e.g., Chen et al., 2019). And they admit that the ceiling effects limited their ability to draw a clear conclusion on the findings using multiple regression analyses (p.659).

Based on our pilot testing, the difficulty levels of our tasks were quite easy for typically developing kindergarten and elementary school children who were a few years younger than our dyslexic participants, suggesting that ceiling effects could be obtained if the tasks were performed by typically developing children at the same age as our dyslexic participants, particularly for tasks that measured accuracy (see Methods).

We have now clarified this issue on p.10.

4. Methodology: Another key flaw in the design of this study is the lack of taking visual perception into consideration, which is considered to be crucial to Chinese reading (e.g., Meng et al., 2011) as well as one of the core deficits of Chinese dyslexia (e.g., Ho et al., 2004).

Meng, X., Cheng-Lai, A., Zeng, B., Stein, J. F., & Zhou, X. (2011). Dynamic visual perception and reading development in Chinese school children. Annals of Dyslexia, 61(2), 161-176.
Ho, C. S. H., Chan, D. W. O., Lee, S. H., Tsang, S. M., & Luan, V. H. (2004). Cognitive profiling and preliminary subtyping in Chinese developmental

dyslexia. Cognition, 91(1), 43-75.

RESPONSE: As discussed in detail on pp.6-7, perceptual expertise with words is different from several existing visual accounts of developmental dyslexia, including the magnocellular-dorsal account and orthographic processing.

Meng et al (2011) focused on the magnocellular theory, in which the visual task was a motion detection task which required participants to judge which one of two patterns had coherently moving dots. This was typically related to the dorsal pathway of the visual system, in contrast to perceptual expertise with words which typically engages the ventral pathway of the visual system. The examination of abilities related to both the dorsal and ventral visual pathways would be an direction for future research.

In Ho et al (2004), the visual measures in this work included three orthographic tasks (left/right reversal, lexical decision and radical position), and contributed to three out of seven subtypes of Chinese dyslexia. These tasks all required participants to have considerable understanding of the writing system, in contrast to perceptual expertise with words that can be performed by individuals who have zero knowledge about the writing system. They also included several additional visual tasks, which focused on judging visual figures, which should highly overlap with the Raven's test in our study that also used visual geometric patterns. Hence our findings have already taken into account the contribution of these abilities to judge visual figures.

5. Methodology (Perceptual fluency): Although the meanings between original stimulus and replacing one were checked, the visual similarities are also matter. Considering the importance of visual modality in Chinese character reading, I expect a prior examination of the stimuli used like Liu, Chen, and Chung (2015) did.

Liu, D., Chen, X., & Chung, K. K. (2015). Performance in a visual search task uniquely predicts reading abilities in third-grade Hong Kong Chinese children. Scientific Studies of Reading, 19(4), 307-324.

RESPONSE: In Liu et al (2015), they used only 4 trials per stimulus type, and only two characters per trial as the target and distractor, and therefore the visual similarity of each pair of characters could be critical and lead to different result patterns.

In our study, we randomized the differences in visual similarity between target-distractor pairs by using 120 trials with a pool of 160 characters commonly recognized by Grade 1 students. With the larger number of trials and characters, the visual similarity between target-distractor pairs was expected to be averaged out.

Furthermore, the stimuli were pre-generated, and all participants used the same stimuli presented with the same trial sequence for each fluency estimate. In this case, the possible influence of any visual similarity of the target-distractor pairs

should not have affected the individual differences in the measured performance in visual fluency with words among our participants.

Reviewer #2: The manuscript presents a study investigating the relation between perceptual expertise with Chinese characters and Chinese word reading in Hong Kong Chinese children with dyslexia. A task measuring perceptual expertise in Chinese character processing, with an adapted visual presentation duration is used to test the individual threshold at which a string of Chinese characters can be discriminated. Individual performance on this task correlated both with speeded and non-speeded reading of Chinese words presented in lists. Hierarchical regression analyses showed that performance on the perceptual expertise task also explained variance in speeded and non-speeded reading after taking into account age, non-verbal IQ, phonological awareness, morphological awareness, rapid automatized naming and performance on the perceptual expertise task but using strings of digits rather than Chinese characters. The authors conclude that perceptual expertise with words plays an important role in Chinese reading and that perceptual training is a potential route to remediation.

2. Is the manuscript technically sound, and do the data support the conclusions?
Based on my reading of the manuscript the experiments seem to have been conducted rigorously and including appropriate sample sizes. Compared to other studies on dyslexia I expected to also see data from a control group, yet conclusions can be drawn based on this dataset alone. I responded that the conclusions are partly supported by the data mainly because there are some aspects of the manuscript which in my opinion could be clarified and further discussed considering the existing literature. There is a large literature on dyslexia and though it is impossible to cover all these in a manuscript it seemed at times that there were some missing links between the perceptual expertise literature (in music etc.) and the dyslexia literature. In the following paragraphs I outline three main points I consider could be improved and some additional minor comments or questions. I enjoyed reading the manuscript and I hope the reviews will be of use to the authors.

1. I am not completely sure that based on the provided description and arguments I have a clear understanding of the (a) concept of perceptual expertise and its limits, (b) to what point perceptual expertise can be considered a potential cause of dyslexia rather than a consequence of less reading experience, and (c) the distinction between perceptual expertise and orthographic processing.
(a) Overall I understand that the authors consider perceptual expertise a domain-specific ability, distinguishing their proposal from other visual theories of dyslexia and supporting this with the reported differences in the contribution of the digit as compared to the character perceptual fluency

tasks. On page 26 when the authors discuss differences in the digit and character perceptual fluency tasks they consider that the unique contribution of the character task to word reading after accounting for performance on the digit task indicates that the character perceptual fluency task reflects a domain-specific skill (suggesting again it is a consequence of reading experience?).

RESPONSE: We agree that visual fluency with characters can be a cause and/or effect of reading impairment. This is discussed on p.26, 1st paragraph, with the suggestion of intervention studies as a way to test the causal role of the visual skill in developmental dyslexia.

While I understand the logic and appreciate the inclusion of the digit version of the task, I think there are potential limitations to this reasoning. Digits are fewer and less visually complex than the Chinese characters, thus it could be expected that acquiring perceptual expertise is less challenging and that the task might be inherently easier. Since similar patterns of correlations were found between these two tasks and reading skills (albeit stronger for the character than the digit span) could it be that the difficulty in the task with characters doesn't tap into a different domain but has better discriminatory power?

RESPONSE: Participants did perform better for the digit fluency task (log duration threshold = 2.58; duration threshold = 381.4ms) than the character fluency task (log duration threshold = 2.87; duration threshold = 747.7ms). However, since the QUEST estimation was performed by a monitor with very high refresh rate (1ms per frame, or 1ms per step of estimation), both duration thresholds were very far from the ceiling performance that can be expressed. Indeed both tasks show very high discriminatory power, as indicated by the high reliability; actually the reliability for digit fluency (0.889) was even higher than that for character fluency (0.849). Therefore it is unlikely that the discriminatory power of either task was constrained.

It is recently clarified that object recognition ability can be explained by both domain-general and domain-specific abilities, both being independent from general intelligence (Gauthier, 2018). Hence character fluency, as a type of visual perceptual expertise, is likely predicted by both domain-specific and domain-general ability, the latter of which is captured by digit fluency. Since both types of fluency involves domain-general visual perceptual abilities, it is also reasonable to observe that they share similar correlation patterns with reading skills, although with different strengths shown by the correlation coefficients. We have now clarified these points on p.27, last paragraph.

Gauthier, I. (2018). Domain-Specific and Domain-General Individual Differences in Visual Object Recognition. Current Directions in Psychological Science, 27(2), 97-102. https://doi.org/10.1177/0963721417737151

A secondary note is that if this skill is considered domain specific then I am not sure it can be reconciled with the results of the visual texture training study that led to improvements in reading (page 5).

RESPONSE: In Meng et al. (2014), it was found that visual texture discrimination training led to improved reading performance. While it is unclear why their training worked, it may be useful to note that texture discrimination typically involves judging basic visual features such as line orientation over a large visual field in the visual periphery. These processes are typically referred to as early visual processes engaging the primary visual cortex (Karni & Sagi, 1991; Schwartz et al., 2002).

In contrast, for perceptual expertise with words, participants typically focus on a few characters presented at the fovea, and these processes are typically referred to as higher or late visual processes engaging the more downstream visual areas corresponding to shape and object recognition. In sum, the two types of tasks are known to be very different.

Because of your question, we realize that it would be misleading to discuss Meng et al (2014) under the paragraphs discussing why perceptual expertise for words might be an important predictor of reading. We have now moved this discussion to p.34, 2nd paragraph, and clarify that they likely address different visual bottlenecks of the reading deficit.

(b) I was not sure whether the authors consider perceptual expertise only a consequence or also a potential cause of dyslexia. The example of the car expert on page 6 would suggest that reading experience alone might result in better perceptual fluency (as a car expert becomes particularly good at discriminating cars because they spend a lot of time looking at cars). In the discussion the authors do suggest that it could be both a cause and consequence of dyslexia, but I am not sure it is clear how it could be a cause.

RESPONSE: Perceptual expertise with words could serve as a cause of reading deficit because the decreased perceptual fluency for characters likely indicates the failure to develop sufficient sensitivity to the diagnostic information of the words and characters during development. This would lead to confusion between words with similar visual features or shapes. In other words, this perceptual deficit might have caused reading difficulty among children with developmental dyslexia. This perceptual bottleneck might lead to an even more significant problem in more advanced reading materials in which the number of similar visual alternatives tends to increase. We have now clarified this on p.25, 1st paragraph.

(c) The authors suggest that perceptual expertise is not related to orthographic processing and I believe they consider that it does not rely on knowing the mappings between characters and linguistic units. On the other hand, the authors acknowledge (page 23) that reading experience can improve perceptual expertise and that "perceptual fluency may become more important when one learns to read more fluently" (page 25). This is also the case for orthographic processing which becomes more important after readers of alphabetic orthographies have moved beyond decoding and start processing multiple letters and larger orthographic units. This can also affect letter processing in tasks that are not reading. Indeed, reading experience allows readers of alphabetic orthographies to also become better at identifying letters in words (word superiority effect) in the Reicher-Wheeler paradigm. I was wondering why this is not considered to be the case in these perceptual fluency tasks. On a related note, on page 5 the authors mention that differences in orthographic depth (additionally to those of character visual complexity) could also lead readers to rely more on the visuo-orthographic structure of the visual codes. Would this also support that this perceptual expertise is not just visual but is related to the mappings between characters and linguistic units and is more like orthographic processing than suggested?

RESPONSE: Thank you for this question such that we can further clarify what we mean. While perceptual expertise with words and orthographic processing are different with different emphases and assumptions, they are not completely unrelated. In some cases, perceptual discrimination of words and orthographic processing might be partially supported by overlapping information, e.g., when some levels of visual diagnostic parts of the words happen to have nice phonemic correspondence. The extent to which they overlap likely depends on multiple factors, such as how the visual codes of a writing system differ from one another, the mapping between the visual code and various types of linguistic information, etc. We have now added a paragraph on p.7 to clarify this issue.

2. The authors link the literature on perceptual expertise and training perceptual expertise. I am not very familiar with this literature so when reading the manuscript I found myself thinking about the multi-element processing aspect of these tasks (also found in RAN tasks) and visual attention span studies that I am more familiar with. Indeed the paradigm used in this study, that uses different presentation durations depending on performance, clearly differentiates it from visual attention span tasks (in which it is set at around 200 ms to allow a single fixation on the string). Nevertheless, it seems that the results of some visual attention span studies could inform the interpretation of those presented in this manuscript and strengthen the discussion. I mention some of those with similar paradigms and others in children with dyslexia learning to read in Chinese (as far as I know the latter use a visual 1-back paradigm) in case they are of interest. In case the authors disagree with this this view perhaps the studies would still allow them to explain more specifically what their own assumptions are and how they differ from other

visual theories of dyslexia. Each of the visual theories of dyslexia mentioned in the manuscript differ greatly (some are visual only, other auditory and visual, other related to magnocellular processing), so I believe it is difficult to set a new theory apart from all of the previous theories without considering the other theories in more depth. They could also discuss whether they consider the aspect of multi-element processing plays a role in their paradigm.
Lobier, M., Zoubrinetzky, R., & Valdois, S. (2012). The visual attention span deficit in dyslexia is visual and not verbal. Cortex, 48(6), 768-773.
Ziegler, J. C., Pech-Georgel, C., Dufau, S., & Grainger, J. (2010). Rapid processing of letters, digits and symbols: what purely visual-attentional deficit in developmental dyslexia?. Developmental Science, 13(4), F8-F14.
Valdois, S., Peyrin, C., Lassus-Sangosse, D., Lallier, M., Demonet, J. F., & Kandel, S. (2014). Dyslexia in a French–Spanish bilingual girl: behavioural and neural modulations following a visual attention span intervention. Cortex, 53, 120-145.
Zhao, J., Liu, M., Liu, H., & Huang, C. (2018). Increased deficit of visual attention span with development in Chinese children with developmental dyslexia. Scientific reports, 8(1), 1-13.
Chen, N. T., Zheng, M., & Ho, C. S. H. (2019). Examining the visual attention span deficit hypothesis in Chinese developmental dyslexia. Reading and Writing, 32(3), 639-662.

RESPONSE: We totally agree that discussing the relationship between perceptual expertise, visual attention span and multi-element processing would strengthen the discussion and clarify how perceptual expertise is different from various visual measurements. Below are the paragraphs we added into the manuscript:

First, in the introduction (p.6, 2nd paragraph), we elaborated on the similarities and differences between the concepts of visual attention span and perceptual expertise. The paragraph is also included below:

> *Second, perceptual expertise with words does not simply reflect visual attention span or general visual attentional skills used to account for developmental dyslexia (11,56,57). These types of measures often appear similar because they both involve simultaneous recognition of multiple visual items presented horizontally and in brief durations. However, they have different assumptions about the underlying skills and the category specificity of these skills. In particular, the critical skills underlying perceptual expertise is shape processing, while that underlying attentional accounts is visual attention. Also, perceptual expertise is often highly specific to a certain object category but attentional skills are not. For example, a car expert can be outstanding in recognizing cars, but only perform with an average performance with birds. This category-specific expertise in object recognition is consistent with the findings that behavioral effects indicating perceptual expertise are typically confined to one's domain of expertise (18,19,58–61). The attentional account of developmental dyslexia, however, is often considered general and observable with different types of visual stimuli such as numbers, shapes or symbols (56,57,62–64).*

Second, in the Discussion section (p.31, 2nd paragraph), we elaborated on how the specific details of measurements would render measures of these two constructs

more similar or different from one another, including the presentation duration of the stimulus, choice of stimulus, visual crowding, and visual span. While the measures of the two constructs could be highly overlapping under certain conditions, we believe that the contribution of perceptual expertise with words in our findings should be beyond that of visual attention span because of the control fluency task with digits, in which the task and visual attention span was identical to that of the fluency task with Chinese characters.

Also, while reading the recommended references suggested by the reviewer, we found that considering the factor of perceptual expertise may inform the seemingly contradictory findings in Ziegler et al (2010) and Lobier et al (2012). The elaborated paragraphs in Discussion are also included below:

> *Although perceptual expertise with words is conceptually different from visual attention span (see Introduction), it is interesting to further consider the details of the tasks measuring them because these specifics would determine how much the actual measurements overlap. For example, measures of visual attention span often apply very brief presentation duration (e.g., 200ms) to measure what participants can perceive without making additional saccades (56,57,64). This would make sure that participants are simultaneously processing several visual elements (or 'multi-element processing') (115). However, in perceptual expertise measures, individuals are highly varied in terms of how fast they can recognize the stimuli (24,36). Hence, only the top experts can demonstrate excellent perception with single visual fixation (and hence meeting the requirement of tasks of visual attention span), while it is possible for other participants to rely on multiple saccades for recognition. Moreover, visual span measures that involve discrimination of highly similar shapes or objects (e.g., using Chinese characters for studying Chinese reading or letters for studying French reading) (116,117), or that involve visual crowding (63) would involve visual discrimination skills central to perceptual expertise, while perceptual expertise measures that present stimuli over a large visual span would reflect skills critical to visual span measures. In the current study, the perceptual fluency task for digits served as a control measure, which required an identical task and visual attention span as that for characters, and therefore the contribution of visual attention span (11,63) should have been partialed out by the perceptual fluency task for digits.*
>
> *Considering perceptual expertise with words may help inform seemingly contradictory findings in studies of visual attention span. Children with dyslexia showed deficits in visual attention span for letters and digit strings but not for symbol strings, and such category selectivity of the deficit has been interpreted as the deficit about symbol-sound mapping that exists for letters and digits only, but not about other visual problems such as the functions of the visual word form system (57). In another study, however, children with dyslexia showed deficit in visual attention span that was similar regardless of whether the stimulus was nameable or non-nameable, which suggested that the deficit is visual but not verbal in nature (56). Considering the factor of perceptual expertise provides a novel angle to these seemingly contradictory findings. Since children typically have much more experience with recognizing letters and digits than with symbols, a possible alternative explanation of findings in Ziegler et al. (57) was that their findings reflected higher degrees of perceptual expertise with letters and digits than with symbols. Further studies may directly test this alternative explanation to clarify this issue.*

Regarding training, there is a visual attention span training program that might also be of interest (COREVA® training program: Valdois et al., 2014) because to my knowledge it includes tasks similar to those suggested by the authors: visual discrimination, string matching. As far as I know a version in Chinese does not exist.

RESPONSE: It was interesting to look into the training tasks of COREVA and we found that the training included the skills of fine-level visual discrimination. We now discuss the relationship between COREVA and perceptual expertise in the discussion, under the section about intervention strategy on p.33, 3rd paragraph:

> *COREVA, a visual attention span intervention for children with dyslexia that received empirical evidence for its efficacy in a case study, did incorporate the skills of fine-level visual discrimination into their training, which is the central perceptual expertise development (117,128). COVERA included three tasks, visual search and discrimination, visual matching and visual parsing. For visual search and discrimination, participants were required to identify targets among distractors in which "their visual similarity (between targets and distractors) was typically high" (p. 130, 117). For visual matching, participants were required to perform a simultaneous matching task – to identify whether two strings of letters, drawings or symbols were identical or not as accurately and as fast as possible. This was highly similar to the perceptual fluency task except for the sequential versus simultaneous presentation of the stimuli, and similar perceptual training protocols have been showed to enhance degrees of perceptual expertise (72,84). For visual parsing, participants were required to search for bigrams or trigrams in a long string of letters as fast as possible. This required participants to recognize a specific combination of letters among other highly similar letters, and again essentially training up one's ability to discriminate between highly similar visual objects. In sum, COVERA often uses letters and highly similar symbols with speeded presentation and/or response, and therefore it essentially improves one's perceptual expertise during training in addition to other skills.*

3. On page 26 the authors consider the possibility of perceptual fluency training and discuss studies focusing on improving visual processing skill and perceptual expertise in other domains. I think that it might also be helpful to explain how a potential improvement in perceptual fluency for character processing (without any training of mappings with linguistic units) could transfer to reading skills and whether/why this training could be superior to a phonological training or a training of character-sound associations.

RESPONSE: Perceptual fluency training may help children with dyslexia improve their reading performance by enhancing their efficiency in discriminating between

visually similar words, in facilitating the association of the words with their linguistic units based on more accurate representation of the visual codes, and alleviating the vicious cycle between reduced perceptual fluency and reduced reading experience. We now elaborate on why perceptual fluency for characters may help improve reading on p.33 2nd paragraph.

The current paper provides evidence to support that perceptual fluency training is a possible intervention strategy that might have unique contribution to improving reading in children with dyslexia. However, it is not our goal to propose that perceptual fluency training is superior to other types of training. Instead, we believe that developmental dyslexia has multiple potential causes, and hence effective intervention likely involves multiple strategies. Discriminating between visual codes effectively is one of the fundamental skills in reading, which supports the development of other multimodal skills underlying effective reading. Hence perceptual fluency training could potentially be combined with other trainings to provide more comprehensive intervention for children with dyslexia. We now clarify this on p.35. 3rd paragraph.

The above were the major points related to the manuscript. I also have some more minor comments or questions that I mention below:
-In the final paragraph on page 7, the authors focus on the differences in processing in reading aloud vs perceptual expertise tasks and suggest that the latter does not involve mapping between characters and linguistic units. Is this really the case? I would assume that the depth of processing is likely to depend on the task (reading aloud, lexical decision, perceptual expertise) but not necessarily that the perceptual expertise task is only visual.

RESPONSE: It is possible that the perceptual fluency task may be influenced by underlying cognitive processes other than visual discrimination, such as phonological or semantic processing. However, on p.7 (last paragraph), the point of our discussion was to compare the perceptual fluency task and the word reading task in terms of their *explicit* task demands: The perceptual fluency task does not explicitly examine abilities in phonology, semantics or speech production. As a result, accurate discrimination of words can be accompanied by incorrect or missing phonological and phonemic representation of the words. In contrast, correct word reading requires accurate visual discrimination, phonology and speech production, and therefore the reading task examines all these abilities. We now clarify this on p.7-8 and on p.29 2nd paragraph.

-On page 9 the authors mention that ceiling effects would result if the tasks were performed by typically developing readers. I was wondering why this is so since in the speeded naming there could still be variability in fluent readers

and the non-speeded task items were chosen so that they would be appropriate for P1 to P5.

RESPONSE: We agree that for some of the tasks, e.g., the speeded naming task, the ceiling or floor effects would not be a problem because the measure can accommodate a wide range of performance.

However, for other tasks that focused on accuracy (e.g., phonological awareness and morphological awareness), ceiling or floor effects could happen. Although the characters were selected based on the curriculum of elementary school students, the task demands would also determine how difficult the actual tasks were. Based on our pilot testing with children with dyslexia and typically developing children who were younger, we found that the task performances of typically developing children were at ceiling, while the performance of children with dyslexia were neither at ceiling nor at floor.

-On page 10, if no group differences were found between participants presented with List 1 and those presented with List 2 this could be reported.

RESPONSE: Performance of the two groups of participants using these two stimulus sets was comparable for all tasks (*ts* $\leq$ 1.42, *ps* $\geq$ .165) except for morphological awareness, in which the performance with two stimulus sets was significantly different, t = -2.45, p = .02. This difference was driven by one of the subtasks, the word production task (t = -3.21, p = .003) but not in the other subtask of concept production (p = .314). We now report this on p.12 2nd paragraph.

-Do the authors consider that performance on the perceptual fluency task would be related to visual or verbal memory?

RESPONSE: Since the stimuli were characters that children commonly should have learnt in Grade 1, it is possible that visual and verbal memory might have contributed to their judgment. We have now clarified this on p.12, last paragraph.

-On page 12, is RAN typically presented as a list rather than a matrix when testing in Chinese or was it presented like this to be more like the word reading?

RESPONSE: In the RAN task, 90 digits were presented along multiple horizontal rows of digits on an A3 paper. This was essentially similar to a big matrix commonly

used in a typical RAN task (Norton et al., 2012). This is now clarified on p.14, 2nd paragraph.

-On page 14, I was wondering whether the measures from the two morphological awareness tasks were correlated. The second task seems quite complex to me and I was wondering how participants performed.

RESPONSE: The two morphological awareness tasks was correlated considerably but not too strongly, r = .396. p = .019, supporting the idea that they tap onto overlapping yet different aspects of morphological awareness skills.

We have now reported the performance of each morphological task during the discussion on the relationship between morphological awareness and word recognition (p.37-38). For the 2nd task, the mean performance was 8.71 out of 15, SD = 3.31, range = 1-15.

-Neither morphological nor phonological awareness correlated with reading. Is this surprising or is it a common finding in reading in Chinese? Were the scores perhaps at floor/ceiling?

RESPONSE: From the revised Table 1, the means of the phonological awareness and morphological awareness tasks were 2.1 SD and 1.6 SD away from the minimum or maximum scores respectively. Hence it is unlikely that the findings were limited by any ceiling or floor effects.

We now added two sections at the end of Discussion (on p.36-39) to discuss the absence of correlation between the constructs and reading scores, and how they relate to the current literature. The paragraphs are also included below:

> *Phonological Awareness and Word Recognition*
> *It is interesting to observe that phonological awareness did not predict either speeded reading or non-speeded reading (Table 2). Phonological awareness refers to the awareness of and access to the sounds of one's language (135,136). While the deficit in phonological awareness is regarded as a major cause of developmental dyslexia for alphabetic languages (3,4), its role in developmental dyslexia in logographic languages such as Chinese is less clear. Earlier studies reported that phonological skills predicted reading performance in typically developing Chinese children (136,137). However, similar findings were not observed in a subsequent large-scale study (138).*
>
> *Findings regarding its contribution to developmental dyslexia were also mixed. For example, while about 30% of Hong Kong Chinese children with dyslexia showed deficits*

in phonological tasks, the unique contribution of phonological awareness did not reach significance when other factors were considered in hierarchical regression models (108). Phonological awareness also failed to distinguish children with developmental dyslexia and typically developing children using logistic regression (88). However, in a longitudinal study using logistic regression, phonological processing during the 3rd year in kindergarten predicted dyslexia outcome a year later in grade 1 in primary school (139). While phonological awareness remains to be important for Chinese language learning for some researchers (88), other researchers considered phonological awareness as less important and not one of the 'core problems' in Chinese developmental dyslexia (108,116).

Our results showed that phonological awareness did not correlate with either speeded or non-speeded reading performance, consistent with the previous findings (107). Given the good reliability of and the absence of ceiling or floor effects in our phonological awareness measure, our findings support the idea that phonological awareness skills may not be an important unique predictor in Chinese developmental dyslexia.

Morphological Awareness and Word Recognition

It is also interesting to observe that morphological awareness did not correlate with either speeded reading or non-speeded reading (Table 2). Morphological awareness refers to the awareness of, the ability to reflect on and the ability to manipulate the structure of the smallest meaningful units, i.e., morphemes, in words (140). In recent years, morphological awareness has been proposed to be a core theoretical construct for explaining Chinese reading abilities (88). Supporting evidence comes from its ability to predict Chinese character recognition in typically developing children (141), to longitudinally predict Chinese character recognition in typically developing children (138), and to distinguish between children with dyslexia from age-matched controls (88; but see 142).

To understand our seemingly inconsistent finding with these evidences, it might be useful to consider the task demands of the morphological awareness measures. In the current study, two subtasks were used. One was the concept production task, which tapped onto how well children understand the morphological structure of the multi-character words. This morphological structure provides very useful hints for the gist of the meaning of multi-character words (e.g., whether it is a type of flower, a type of fish or a type of machine), and therefore enhancing the semantic transparency of the words (138). This is a relatively easier task (mean = 16.0 out of 19 points, SD = 3.47, range = 2-19) and is commonly used with younger children. We adopted this easier task to pick up the variance in morphological awareness in relatively weaker readers among children with dyslexia.

The other task was a word production task, which was relatively more difficult (mean = 8.71 out of 15 points, SD = 3.31, range = 1-15) and was often adopted for older children. To perform well, one needs to fulfill two task demands: to differentiate whether the homophones in different two-character words were the same or different characters, and whether these homophones carried the same meaning or not. To

achieve these, it is helpful to retrieve and discriminate between the visual codes of the target character based on the pronunciation. Given the high number of homophones in Chinese language, the ability to discriminate between the retrieved visual codes becomes even more helpful. This hypothesis is supported by the significant correlation between morphological awareness and perceptual fluency for characters (Table 2).

In other words, the morphological awareness measures in the current study included two tasks, each tapped onto different aspects of morphological skills with different difficulty levels. Our participants were heterogeneous in terms of their abilities in word reading and in different aspects of morphological skills, as demonstrated with the huge range of performance of each task. It is possible that the previously observed relationship between morphological awareness and reading could be observed more easily in a relatively more homogeneous sample (e.g., among typically developing children; e.g., 138,141), or in categorizing participants into the dyslexic and control groups which was more robust to the heterogeneity of the data (88). This could potentially resolve the seemingly inconsistent findings and should be examined in further studies.

-Morphological awareness correlated with performance on the perceptual fluency tasks. Would this also indicate that performance on the perceptual fluency tasks is something more than visual processing or is the common variance related to something else?

RESPONSE: As discussed above, given that the word production task was based on homophones, one would benefit from the ability in discriminating the visual codes of the two-character words. Hence it is reasonable to observe a significant correlation between morphological awareness and perceptual fluency with characters.

-Table 1. It was not clear to me what each measure reflects (accuracy, speed sec-ms) and whether in these tasks there are minimum/maximum minimum possible scores. If there aren´t actual minimum maximum scores, then perhaps providing the range of scores could be helpful for the reader. I also consider that presenting the raw threshold values from the perceptual fluency tasks (additionally to the log transformed values) could be useful so the reader can more easily interpret the numbers.

RESPONSE: In Table 1, we have now added the range and possible range (maximum and minimum scores, if any) of each measure. We have also directly transformed the log values in the table into threshold values in milliseconds and added them into the table for easier interpretations of the numbers.

-Tables 3 and 4. I am not sure I understand what the capital B stands for.

RESPONSE: The *B* and *β* stand for unstandardized and standardized beta respectively. There was a typo in the original version, in which the bold and non-italic 'B' should be *β*. These are now clarified and corrected on Table 3 and 4.

3. Has the statistical analysis been performed appropriately and rigorously? It seems that the analyses have been conducted appropriately although some additional information on the tests used and distribution of the data could be presented. I am not sure it is mentioned but I assume that data for each variable were normally distributed since parametric tests have been used. I am not an expert on regression models but based on my experience I was a bit surprised that it was possible to fit a model including so many variables with only 35 participants without overfitting.

RESPONSE: Our data generally fit the 3 assumptions of regression well. First, regression assumes a normal distribution of the residuals of the regression, which could be indicated by the general conformity of the expected cumulative probability and the observed cumulative probability in our data, as shown on the P-P plots:

Speeded reading:

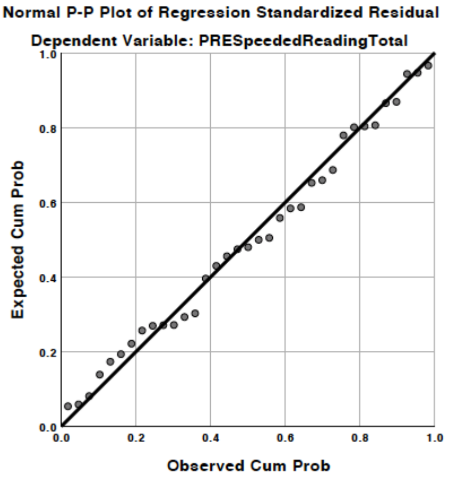

Non-speeded reading:

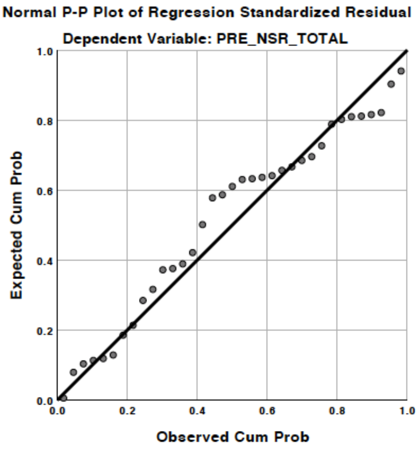

Second, regression assumes homoscedasticity, or whether the residuals are equally distributed. This can be reflected by the widely distributed data points in the following plots with regression standardized residual against regression standardized predicted values:

Speeded reading:

Non-speeded reading:

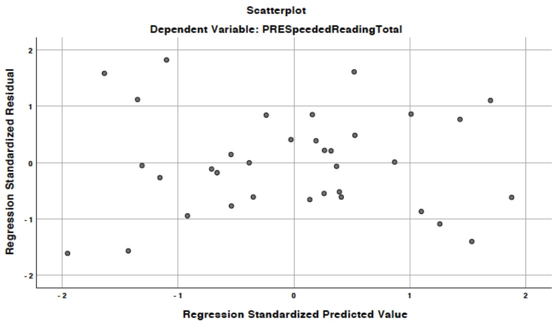

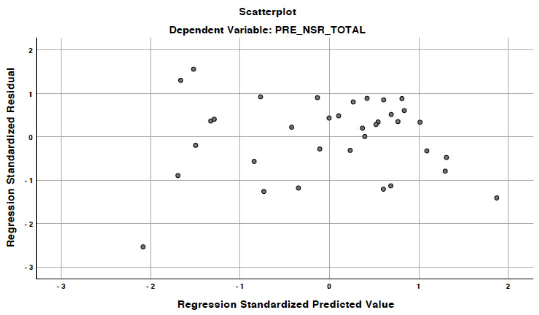

Third, regression assumes the absence of multicollinearity of the predictors, which can be indicated by the relatively small variance inflation factor (VIF) values (ranged 1.14 – 2.68, which is far below a suggested reference level of 10.00).

The overfitting issue is responded in detail below.

Is it the case that there is more than one observation per participant for the reading scores?

RESPONSE: No, there was only one observation per participant for each reading task.

Is there a way the authors could check for overfitting?

RESPONSE: We examined the issue of overfitting with predictive R-squared. This method removes a data point from the dataset, generates the regression model and evaluates how well the model predicts the missing observation. Large discrepancy between the original R-squared value and the predictive R-squared value indicates that the model does not predict new observations as well as it fits the original dataset, and therefore suggests that overfitting might have occurred in the regression model. For example, an original R-squared of 0.5 and a predictive R-squred of 0.05 would mean that the main contribution to the original R-squared involves overfitting.

We found that for speeded reading, the original R-squared was 0.707 and the predictive R-squared was 0.426. For non-speeded reading, the original R-squared was 0.590 and the predictive R-squared was 0.319. There was a considerable difference, yet the results were still largely generalizable.

I was also wondering whether, when adding perceptual fluency for characters in the speeded and non-speeded reading models it was possible to check if adding this variable significantly improved the model overall (additionally to checking the variance it explained after it was added).

RESPONSE: As described in the Result section (p.21, 2nd paragraph), both models for speeded reading and non-speeded reading was significantly improved after adding the variable of perceptual fluency for characters:

For speeded reading, ΔR^2 = .053, $\Delta F(1,27)$ = 4.82, p = .037.

For non-speeded reading, ΔR^2 = .096, $\Delta F(1,27)$ = 6.35, p = .018.

4. Have the authors made all data underlying the findings in their manuscript fully available?
Yes, the data is available.

5. Is the manuscript presented in an intelligible fashion and written in standard English?
Yes.

Reviewer #3: The introduction is clear and easy to follow. The analysis of results appears to be appropriate, and it was good to see reports on reliability of all tasks. My comments are mostly about discussion points, interpretations, and some lack of details.

Alternative interpretations of your results:

You should absolutely discuss alternative interpretations of your data. For example, in your perceptual fluency task, you manipulate the duration of the target (characters or digits) depending on performance and find that poorer readers need more time to process characters. While you interpret this as a perceptual expertise deficit, would this ever have been unexpected even from the standpoint of other theories of dyslexia, as slow reading is one of the characteristics of the disorder in the first place? If they read poorly, the characters will have disappeared before they can read them all successfully and hence, they cannot match them well unless they are shown for a longer time. What if this is e.g. because poorer readers take longer converting each character to a phonological code? Or they have poorer verbal working memory, which could play a part in this task? Or they have problem with crowding or object recognition regardless of experience, as characters are likely more self-crowding (crowding occurs between the parts of an object, see

Martelli et al. 2005) and more visually complex than numbers? Etc.

Martelli, M., Majaj, N. J., & Pelli, D. G. (2005). Are faces processed like words? A diagnostic test for recognition by parts. Journal of Vision, 5(1), 6-6.

RESPONSE: We agree that being a 'poor reader' is a major characteristic, and essentially one of the major assessment criteria of Chinese dyslexia. Our goal is to take a step further to ask *why* these children read poorly and examine whether perceptual fluency is one of the factors underlying their poor reading performance.

As we now elaborated on p.29, 2nd paragraph, existing associations between the characters and linguistic units (e.g., phonological codes) or verbal working memory could contribute to performance of the perceptual fluency task. However, it is important to note that the perceptual fluency task does not require linguistic processing (unlike a word reading task), nor does it tax heavily on working memory (with merely a 500-ms gap between the study and test). For example, one could correctly respond to a trial in the perceptual fluency task with correct, incorrect or non-existing visuo-phonological association. Also, the contributions of other major cognitive abilities associated with dyslexia, including phonological awareness and other tasks that heavily involved verbal memory (e.g., the morphological awareness tasks) have been partialled out in the multiple regression analyses, so it is unlikely that the role of perceptual fluency in word reading is solely explained by these factors.

For the issue of visual crowding, past studies have shown that perceptual expertise typically leads to alleviation of visual crowding that is specific to the expert object category, but not with other untrained shapes (Wong & Gauthier, 2012; Wong & Wong, 2016). Importantly, the perceptual fluency task with digits were identical to that with characters in terms of how the stimuli were spatially presented, and therefore general visual crowding effects should have been partialled out by the perceptual fluency task with digits. Furthermore, perceptual fluency with characters is critically different from visual crowding. In a visual crowding task, one is typically told to report the target and ignore the distractors nearby (Bouma, 1970; Pelli & Tillman, 2008; Legge, 2008), while the perceptual fluency task requires the participant to attend to all characters. This task difference is critical, because training focused on visual crowding does not lead to improved reading (Chung, 2007), while training focused on recognition of all of the presented letters do (Chung, 2004; see also Wong & Wong, 2016). These suggest that the development of perceptual expertise involves skills to address the visual crowding problem, but visual crowding per se cannot fully explain the visual skills involved in perceptual expertise. This is now clarified on p.30, 3rd paragraph.

Category specificity:

As you pointed out in the beginning of the article (p. 6), perceptual expertise is often highly specific to a certain object category. Would you expect a perceptual expertise deficit in dyslexia to be specific to words/characters? If so, why? If not, how would you expect this problem to manifest for other visual objects? The current evidence for this is mixed. E.g. Gabay et al. found problems in dyslexic readers for faces, an expertise category, but not for cars, leading these authors to suggest that: "…DDs' impaired performance on face and word stimuli can be accounted for by difficulties in learning or gaining perceptual expertise (and the ability to make finegrained discrimination among a group of homogeneous exemplars)." Sigurdardottir et al. (2018) found a problem with faces but not novel objects, again in accordance with a visual expertise account, where they: "…speculate that reading difficulties in dyslexia are partially caused by specific deficits in high-level visual processing, in particular for visual object categories such as faces and words with which people have extensive experience." Sigurdardottir et al. (2019) again found problems with faces, but they found this regardless of experience with faces (own vs. other-race faces), leading them to say that: "Visual problems in dyslexia are not demonstrably dependent on visual experience."

Gabay, Y., Dundas, E., Plaut, D., & Behrmann, M. (2017). Atypical perceptual processing of faces in developmental dyslexia. Brain and language, 173, 41-51.

Sigurdardottir, H. M., Fridriksdottir, L. E., Gudjonsdottir, S., & Kristjánsson, Á. (2018). Specific problems in visual cognition of dyslexic readers: Face discrimination deficits predict dyslexia over and above discrimination of scrambled faces and novel objects. Cognition, 175, 157-168.

Sigurdardottir, H. M., Hjartarson, K. H., Gudmundsson, G. L., & Kristjánsson, Á. (2019). Own-race and other-race face recognition problems without visual expertise problems in dyslexic readers. Vision research, 158, 146-156.

RESPONSE: While the word reading deficit of some children with developmental dyslexia may be caused by a failure to develop sufficient perceptual expertise with words, it is unclear to what extent such failure is specific to the object category of word recognition vs. reflects general deficits in high-level visual recognition abilities. Recent studies have reported impaired performance in object recognition in children with developmental dyslexia, suggesting the deficits in object recognition is not limited to words but can be generalized to other object categories (e.g., Gabay, 2017; Sigurdardottir, 2018; 2015; 2019). However, these findings are inconsistent in how 'general' the higher-level visual impairment is, i.e., whether one should expect it to be observed in all object categories in general (e.g., Sigurdardottir et al., 2015), or only in some (Gabay et al., 2017). Also, it is unclear how these visual deficits in other object categories are related to their word reading abilities, e.g., whether these general high-level visual deficits *cause* word reading deficits or whether these general deficits are independently caused by other conditions of the participants. This question would be directly relevant to the formulation of intervention strategy

(see below), e.g., whether the intervention should be specific to words or general to multiple object categories. The current findings cannot directly answer this important question, and it should be clarified by future studies. We have now discussed this in the Discussion on p.28, 2nd paragraph.

No control group:

On page 9, you say: "Note that we were interested in investigating the variability within children with dyslexia such that the difficulty level of the tasks was designed to be appropriate for their ability. Ceiling effects could easily result if the tasks were performed by typically developing children. Hence comparing the role of perceptual expertise with words in normal readers and readers with dyslexia was out of the scope of this study." You have Raven's, which should cover a wide range of abilities, perceptual fluency, which by definition covers a wide range of abilities as it uses a staircase procedure, and RAN and speeded reading, both of which measure time which again should cover a wide range of abilities. I can see how non-speeded reading, phonological awareness, and possibly morphological awareness might have ceiling effects in a typical sample, but you have lots of tasks that would be fine for a control group.

RESPONSE: We agree that having a control group would be fine for some tasks but not for the others. Since one of our goals was to explore the correlations with and unique contribution of perceptual fluency with characters on word reading on top of other known factors associated with dyslexia, it is important to have *all* these cognitive tasks off ceiling and floor for all participants included in these analyses. As described above, we performed pilot testing with children with dyslexia, and found that tasks that were off ceiling and floor for children with dyslexia could easily lead to ceiling effects for typically developing children. Hence we decided to focus on the children with dyslexia for this current study.

Adding details:

Some details in the procedure are missing. E.g. on page 11, you say that you used a premask in the perceptual fluency test. What kind of mask, what were its properties?

RESPONSE: The mask presented before and after the stimulus was a greyscale image created by segments of prints (e.g., letters and digits). It is illustrated in Figure 1, and is now clarified in the text on p.13.

You say that you showed four characters. Were they always aligned vertically or did that vary?

RESPONSE: The character sequences were always arranged horizontally. It is illustrated in Figure 1 and is now clarified on p.13.

Was the choosing of the to-be-replaced character location (first, second, third, fourth) random, counterbalanced, other?

RESPONSE: The position of the replaced character in the distractor was counterbalanced. It is now clarified on p.13.

Why did you use characters 0-9 except 1 in the perceptual fluency task, but digits 2, 4, 6, 7, and 9 in RAN?

RESPONSE: In perceptual testing with digits, it is common to avoid using ‘1’ because it is simply a vertical stroke, which easily stands out from the other alternatives. It is now clarified on p.11, 2nd paragraph.

For RAN, we followed the tradition of using 2,4,6,7,9 in the standard testings (Norton et al., 2012).

Was the choice of logarithm of the duration thresholds based on how these perceptual fluency tasks have been previously run with e.g. English words, musical notation etc., or was it idiosyncratic for this study, and then why?

RESPONSE: The logarithmic transformation of a non-linear relationship between two variables is a common strategy for producing more interpretable results (e.g., Chung, 2004).

For example, a 100ms response time improvement from 200ms to 100ms means a significant improvement (a 100% change), while that from 2100ms to 2000ms is relatively negligible (a 5% change). Transforming the response time measure with logarithm would linearize the relationship between duration threshold and performance. For example, the same 100ms improvement between 200ms and 100ms would become 0.30 in the log scale, a much larger difference than that between 2100ms and 200ms (0.021). This is a commonly applied strategy to deal with non-linear relationship between variables (e.g., Chung, 2004), which makes the findings more interpretable. This is now clarified on p.14, 1st paragraph.

In phonological awareness, you talk about tone, can you briefly explain to non-Chinese speakers what you mean? Or was it just three different people with three different voices that read the characters?

RESPONSE: Cantonese is a tonal language, and the tone of a character is indicated by the number (e.g., ‘1’ denotes a high flat tone; ‘2’ denotes a rising tone; ‘3’ denotes a flat mid-pitch tone lower than ‘1’; etc.). Notably, identical onset phoneme and rime pairing with different tones could have distinct meanings (e.g., kei1, kei3, kei4 and kei5 may mean abnormal, to hope for, a flag, and to stand up respectively; and there are additional homophones with other meanings too). In the oddball task under phonological awareness, the tone could either be shared or all different among the 3 characters within each trial. We have now clarified these on p.15, last line.

7. PLOS authors have the option to publish the peer review history of their article (what does this mean?). If published, this will include your full peer review and any attached files.

If you choose “no”, your identity will remain anonymous but your review may still be made public.

Do you want your identity to be public for this peer review? For information about this choice, including consent withdrawal, please see our Privacy Policy.

Reviewer #1: Yes: Li-Chih WANG

Reviewer #2: No

Reviewer #3: No

[NOTE: If reviewer comments were submitted as an attachment file, they will be attached to this email and accessible via the submission site. Please log into your account, locate the manuscript record, and check for the action link "View Attachments". If this link does not appear, there are no attachment files.]

While revising your submission, please upload your figure files to the Preflight Analysis and Conversion Engine (PACE) digital diagnostic tool, https://pacev2.apexcovantage.com/. PACE helps ensure that figures meet PLOS requirements. To use PACE, you must first register as a user. Registration is free. Then, login and navigate to the UPLOAD tab, where you will find detailed instructions on how to use the tool. If you encounter any

issues or have any questions when using PACE, please email PLOS at figures@plos.org. Please note that Supporting Information files do not need this step.
