# Supplementary material for: Perceptual expertise with Chinese characters predicts Chinese reading performance among Hong Kong Chinese children with developmental dyslexia
Source: PLoS One. 2021 Jan 22;16(1):e0243440. doi: 10.1371/journal.pone.0243440 (PMC7822259; doi:10.1371/journal.pone.0243440)
Supplement: Attachment — Submitted filename: ResponseLetter_v1.docx [file pone.0243440.s001.docx]

Dear Prof. Protopapas,

Thank you for your constructive comments. We have now clarified our wordings hoping to minimize confusion among researchers from different backgrounds.

We sincerely hope that now you would find the manuscript suitable for the review process.

Thank you very much for your time.

Yetta Wong & Alan Wong

*********************

**PONE-D-20-07055
Visual discrimination skills predict Chinese reading performance among Hong Kong Chinese children with developmental dyslexia
PLOS ONE

Dear Dr. Wong,

Thank you for submitting your manuscript to PLOS ONE. To save everyone's time, I screen incoming manuscripts before sending them out for review. There are two issues that prevent me from considering this submission further as it stands:**

**First, your submission does not adhere to the data sharing guidelines of PLOS ONE. You only indicate that data will be posted after acceptance and that there will be restrictions to access. However, minimal data must be included with the manuscript, provided with submission, unless extraordinary (fully justified) circumstances prevent this, and any foreseen restrictions to accessing all the data upon publication must be fully documented and justified. Please consult the PLOS ONE data policy (**[**https://journals.plos.org/plosone/s/data-availability**](https://journals.plos.org/plosone/s/data-availability)**) to ensure full compliance before resubmission.**

RESPONSE: Thank you for this suggestion. During the preparation of the dataset, we identified errors in a small subset of manually input data, which led to minor adjustments of the reported numbers (mostly in the tenths and hundredths decimal places). These did not affect the major pattern of the results. We have now corrected these errors with tracked changes. We have also included the dataset ready to be shared upon acceptance of the manuscript, which is available here:

https://osf.io/dcth6/?view_only=5ab8b6d793b54f24b5fcbf5471bed83c

**Second, I am worried about the terminology used in your submission, and in particular with respect to the critical task, which may turn out to be misleading for readers in the field, as it is used in the title as well as the body of the manuscript. Specifically, you talk about "visual discrimination" where in fact you task is neither "visual" in the commonly encountered sense nor "discrimination". Of course it is visual in the sense that one must see the details of the characters correctly in order to perform the task. But the term "visual" is typically reserved for skills that are not specific to certain kinds of stimuli, as this is commonly understood to be a generic designation. Instead, your findings concern the visual processing of characters, and indeed beyond performance with digits, therefore squarely excluding any generic "visual" component and instead concerning the processing of orthographic material. As expected based on the effects of experience and as your previous published work demonstrates, perceptual expertise with characters covaries with reading skill, so there is nothing surprising or problematic about that. Character recognition expertise is a marker of reading proficiency, and it would be very surprising if dyslexia was not associated with more difficulty in recognizing, retaining, and matching characters.**

RESPONSE: In the initial draft of the manuscript, we thought some of the reading researchers may not care about the ‘perceptual nature’ of this task and therefore

we described the task as ‘visual’ for simplification.

Based on your comments, we realized that this simplification could actually cause confusion for other researchers who care about these differences.

Now, we called this factor ‘perceptual expertise with words’ (or ‘perceptual expertise with characters’ when referring to Chinese characters) throughout the manuscript, and the task ‘perceptual fluency task’.

**in your study, as I understand it, the task concerns the identification of characters and indeed includes a memory component, further distancing from what might be considered a strictly "visual" task, as participants must hold the character sequence in memory in order to respond in the two-alternative-forced-choice setup of the task. Which brings me to the second term, namely "discrimination". In a discrimination task one typically must distinguish between two or more stimuli (or aspects thereof), usually (but not always) responding whether they are the same or different. Of course more complex discrimination arrangements do exist, but I find it difficult to classify your task as discrimination when the participant must remember a character (or digit) sequence and subsequently match it to a displayed array of characters. This sounds like an identification task to me. If you disagree, please justify your choice of terms in the revision, so that reviewers will be clear about how the terms are used.**

**If the above comments indicate that I have misunderstood your task or some critical aspect of it, please clarify your description to avoid similar misunderstanding by others. Finally, please note that "novelty" or perceived theoretical importance are not considered as publication criteria for PLOS ONE, so you do not need to feel any pressure to establish any of these; please set up the rationale and terminology of the study to be more precisely in line with the tasks used.**

RESPONSE: As described above, we now avoid describing the underlying factor as ‘discrimination’ by referring it to ‘perceptual expertise with characters’. Hopefully this would be more accurate and minimize confusion for researchers with different backgrounds.
