# Supplementary material for: Perceptual expertise with Chinese characters predicts Chinese reading performance among Hong Kong Chinese children with developmental dyslexia
Source: PLoS One. 2021 Jan 22;16(1):e0243440. doi: 10.1371/journal.pone.0243440 (PMC7822259; doi:10.1371/journal.pone.0243440)
Supplement: Attachment — Submitted filename: ResponseLetter_R3_v2.docx [file pone.0243440.s003.docx]

Dear Professor Protopapas,

Thank you for your action letter. We are very grateful for getting the constructive feedback, especially the part about ‘reading predicts reading’ such that we have a chance to address this concern directly.

We have now responded to the comments below and revised the manuscript accordingly. We hope you will find the manuscript suitable for publication.

Thank you again for your attention.

Yetta Wong & Alan Wong

*******************************************************

**PONE-D-20-07055R2
Perceptual expertise with Chinese characters predicts Chinese reading performance among Hong Kong Chinese children with developmental dyslexia
PLOS ONE

Dear Dr. Wong,

Thank you for submitting your manuscript to PLOS ONE. The reviewers note that you have made an admirable effort to address the comments of the first round of reviews, resulting in an improved manuscript, and I agree. Both reviewers are positively inclined to the eventual acceptance of your manuscript for publication at PLOS ONE, as am I. However, Reviewer #3 makes a number of critical observations, with which I agree, and which I believe you should fully address in the manuscript before it can be accepted. I am therefore inviting you to submit a revision in which you address these issues.**

**In particular, it seems to me that you greatly overplay a supposed theoretical difference between "perceptual fluency" and reading, while you downplay the role of short-term verbal memory in your "perceptual fluency" task.  I agree with Reviewer #3 that your study essentially shows that "reading predicts reading" (although measured in different ways and perhaps stressing different aspects). This does not mean that the study is not useful or cannot be accepted for publication, however as there are at least two of your colleagues (Reviewer #3 and myself) who are of this opinion, it is reasonable to imagine that there are probably more out there, so it seems wise to take this point more seriously in the manuscript in order to maximize your impact. Your perceptual fluency task is a masked word identification task; and for all of the reasons you mention that concern word specificity as an aspect of expertise, for some of us this means that this is basically a word reading task.  Note that tasks manipulating word presentation duration are not unheard of in the word recognition literature; indeed there are recent attempts to use such tasks to assess "word reading automaticity" (Roembke et al., 2019;** [**https://doi.org/10.1037/edu0000279**](https://doi.org/10.1037/edu0000279)**). I believe that there are some problems with that approach as well, but the point is that more readers might share the view of Reviewer #3 regarding what it is you are measuring.**

RESPONSE: Thank you for raising this concern again such that we have a chance to address this directly in the manuscript. We now elaborated in detail why we respectfully disagree that our findings simply showed ‘reading predicts reading’, and why the perceptual fluency measure can emphasize one’s ability in visual discrimination given the possible involvement of subsequent linguistic and memory processes in task performance. This discussion is now on p.29 and is also included below:

*Task Demands of The Perceptual Fluency Task and Reading*

*Although the perceptual fluency task focused on the ability to visually discriminate between words, it is worth noting that this task is unlikely a purely visual task. For example, since the stimuli were real-world Chinese characters, existing associations between the visual form of the characters and other linguistic representations (e.g., phonological, morphological or semantic units) might have contributed to their performance. Also, the stimuli were Grade 1-level characters that participants should have learned previously, and therefore visual and verbal memory might have contributed to their judgment. Do these make the visual fluency task essentially a ‘reading’ task such that the findings reported here simply showed ‘reading predicts reading’?*

*Several considerations would help inform this discussion. First, one should clarify the exact definition of ‘reading’ that one adopts. If ‘reading’ refers to recognition of the visual form of the word only and does not consider any subsequent linguistic processes, then this would be highly similar to the visual discrimination ability measured by the perceptual fluency task. Alternatively, if ‘reading’ requires one to correctly extract the pronunciation and/or the semantics of the words based on the visual code, then regardless of whether the ‘reading’ is done aloud or silently, this is critically different from the perceptual fluency task which did not explicitly examine the accuracy of responses in these linguistic domains.*

*Second, with the use of real-world and learned Chinese characters, one might have activated existing associations between the visual form of the characters and other linguistic units during the perceptual fluency task. In this sense, the perceptual fluency task appears to be highly similar to ‘reading’ since linguistic information can potentially play a role. In response to this, one should consider the fact that word recognition involves many complicated cognitive processes that tend to interleave with each other. Readers likely rely on multiple processes to solve most tasks and therefore a single process cannot be truly isolated. To investigate a specific cognitive process, a more effective way is to use specific task designs combined with control measures to emphasize the interested process and minimize the contribution of others.*

*For the perceptual fluency measure, visual discrimination ability was emphasized using a sequential matching paradigm with speeded presentation. To perform well, one’s perceptual analyses of the character sequence within the brief presentation time must be sufficiently adequate to differentiate it from the distractor, otherwise any subsequent linguistic processes could be error-prone and thus not helping. It is also worth noting that our use of four-character sequences imposed a much higher visual perceptual demand compared with other word recognition tasks that also employed speeded presentation with one single word (e.g., 112).* *When the perceptual analyses are adequately performed, there is no way to stop one from activating the subsequent linguistic processes associated with the characters. However, the contribution of these linguistic processes to one’s performance has been minimized in the current design in several ways. One is that the task did not explicitly examine these linguistic responses, as discussed above, and therefore their existence and accuracy were not considered. This is in contrast to any ‘reading’ task that requires correct pronunciation or semantic retrieval of the words. The other is that, under brief presentation of the characters, the room for activating the linguistic processes is largely constrained. For example, with a presentation time of 300ms or below, it is not straightforward even for adult native readers to retrieve the verbal labels of all characters, at least within one’s awareness, before the characters disappear. In other words, with a faster presentation duration of the characters, the role of visual discrimination becomes more important while that of subsequent linguistic units becomes more limited. The last one is that the use of random character sequences removed any semantic contexts such that characters of any pronunciation or meaning could fit in well, and therefore the usefulness of linguistic information to inform correct responses was largely reduced.*

*Finally, given the use of learned characters, participants might have retrieved their verbal labels, that might have helped the retention of the stimuli during the time gap between the study and test images. In this case, one might suspect that the current findings simply reflected verbal short-term memory, as a common product of ‘reading’, instead of perceptual fluency with characters. However, this is unlikely because of several reasons. First, the time gap between the study and test images was merely 500ms. This largely limited the amount of memory decay during the retention period (113), and therefore one’s working memory ability should have minimal influence on his or her performance. Second, while we acknowledge the potential contribution of verbal labels to one’s performance, this was shared by the perceptual fluency task with digits and should therefore be partialled out in the multiple regression analyses.*

*In sum, the perceptual fluency task is not simply another ‘reading’ task that requires correct access to linguistic information. While the perceptual fluency task may involve many cognitive processes, the critical task demand that determines one’s performance is visual discrimination, especially when the stimulus is presented with brief durations. The contribution of other cognitive abilities, including that of short-term memory, is further tempered with the control measures, in particular the perceptual fluency with digits, which should have partialled out the contribution of other major cognitive abilities known to contribute to reading performance with dyslexia.*

**Furthermore, the need to retain a set of 4 words so that it can be matched after 500 ms suggests that verbal short-term memory may carry most of your effect, as noted by Reviewer #3. It seems reasonable to imagine that your participants performed the task by (verbally) remembering the words, or at least by partial support from verbal memory, as you also acknowledge in the manuscript. The only way to ensure this is not the case is to use unfamiliar visual stimuli that have no verbal association, but this is obviously impossible by definition when you are specifically interested in perceptual expertise with words. (This again goes back to the idea that your perceptual expertise is a kind of a reading task.) The potential involvement of verbal memory is also an issue with much VAS research, especially the commonly used full-report version of VAS, and there are indications that when this aspect of the task is removed then the association with reading may diminish or disappear (Banfi et al., 2018;** [**https://doi.org/10.1371/journal.pone.0198903**](https://doi.org/10.1371/journal.pone.0198903)**).  Again, this does not invalidate your study or your manuscript, but it does suggest that some of your claims need to be greatly tempered, with these alternative views taken into serious consideration in the manuscript.**

RESPONSE: We now elaborated in detail why it is unlikely that verbal working memory carried most of our effects because of two reasons, namely a small time gap that limited the amount of decay in working memory, and also the control task of perceptual fluency with digits that could share a similar contribution of working memory to performance, which should have been partialled out in the multiple regression analyses.

The discussion is now on p.31, last paragraph, which has been included in the response above. It is also included again below for easy reference:

*Finally, given the use of learned characters, participants might have retrieved their verbal labels, that might have helped the retention of the stimuli during the time gap between the study and test images. In this case, one might suspect that the current findings simply reflected verbal short-term memory, as a common product of ‘reading’, instead of perceptual fluency with characters. However, this is unlikely because of several reasons. First, the time gap between the study and test images was merely 500ms. This largely limited the amount of memory decay during the retention period (113), and therefore one’s working memory ability should have minimal influence on his or her performance. Second, while we acknowledge the potential contribution of verbal labels to one’s performance, this was shared by the perceptual fluency task with digits and should therefore be partialled out in the multiple regression analyses.*

**Let me point out that PLOS ONE does not require that your study leads to a definitive novel theoretical contribution; it only requires that your conclusions are supported by the data. Therefore, tempering or adjusting your conclusions to allow for different conceptions of the tasks does not diminish the potential of your manuscript for acceptance; instead, it increases it. In this sense I want to stress that these comments are purely constructive.**

**Finally, I would also like to point out a couple of additional minor comments to take into account in your revision:**

**On p. 3, phonological awareness has nothing to do with perception of the sounds, it is a meta-linguistic skill that concerns conscious awareness and manipulation.**

RESPONSE: Perception and recognition of sounds is the most primitive level of different levels of phonological awareness as defined in Stahl and Murray (1994). We have now rephrased this sentence as the following on p.3:

*…impaired ability to perceive, access and manipulate sounds of spoken words in awareness (“phonological awareness”, 3–5)*

**On p. 9 you argue against a "spatial" interpretation partly on the basis that no 3rd dimension is involved. I think this is a misunderstanding. Two-dimensional space still concerns spatial relations, therefore, two-dimensional visuospatial skills are conceivable and measurable. There is no requirement of depth for something to be considered "spatial".**

RESPONSE: We have now clarified that perceptual expertise does not involve *the type of* visuospatial abilities used to investigate developmental dyslexia before (p.8, last paragraph).

Also, we have also included a remark that perceptual expertise measures also involve two-dimensional visuospatial arrangement of the words (p.9, 1^st^ paragraph).

**On p. 15 you describe the PA task as involving characters but PA is a purely oral skill that has nothing to do with characters or any other aspect of the written language. I imagine that here you mean "syllables" or "words" rather than characters (after all, characters are orthographic entities, they cannot be spoken, only visually presented)**

RESPONSE: In English, characters are orthographic entities that cannot be spoken. In Chinese, however, each character corresponds to a syllable such that they can be visually or orally presented. We have now clarified that the orally presented characters were ‘Chinese characters’, and each character corresponded to a syllable on p.15, last 2 paragraphs.

**Please submit your revised manuscript by Nov 26 2020 11:59PM. If you will need more time than this to complete your revisions, please reply to this message or contact the journal office at** [**plosone@plos.org**](mailto:plosone@plos.org)**. When you're ready to submit your revision, log on to** [**https://www.editorialmanager.com/pone/**](https://www.editorialmanager.com/pone/) **and select the 'Submissions Needing Revision' folder to locate your manuscript file.  

Please include the following items when submitting your revised manuscript:**

- **A rebuttal letter that responds to each point raised by the academic editor and reviewer(s). You should upload this letter as a separate file labeled 'Response to Reviewers'.**
- **A marked-up copy of your manuscript that highlights changes made to the original version. You should upload this as a separate file labeled 'Revised Manuscript with Track Changes'.**
- **An unmarked version of your revised paper without tracked changes. You should upload this as a separate file labeled 'Manuscript'.**

**If you would like to make changes to your financial disclosure, please include your updated statement in your cover letter. Guidelines for resubmitting your figure files are available below the reviewer comments at the end of this letter.

If applicable, we recommend that you deposit your laboratory protocols in** [**protocols.io**](http://protocols.io/) **to enhance the reproducibility of your results. Protocols.io assigns your protocol its own identifier (DOI) so that it can be cited independently in the future. For instructions see:** [**http://journals.plos.org/plosone/s/submission-guidelines#loc-laboratory-protocols**](http://journals.plos.org/plosone/s/submission-guidelines#loc-laboratory-protocols) **We look forward to receiving your revised manuscript.

Kind regards,

Athanassios Protopapas
Academic Editor
PLOS ONE


[Note: HTML markup is below. Please do not edit.]

Reviewers' comments:

Reviewer's Responses to Questions**

**Comments to the Author

1. If the authors have adequately addressed your comments raised in a previous round of review and you feel that this manuscript is now acceptable for publication, you may indicate that here to bypass the “Comments to the Author” section, enter your conflict of interest statement in the “Confidential to Editor” section, and submit your "Accept" recommendation.**

**Reviewer #2: All comments have been addressed**

**Reviewer #3: (No Response)**

**2. Is the manuscript technically sound, and do the data support the conclusions?

The manuscript must describe a technically sound piece of scientific research with data that supports the conclusions. Experiments must have been conducted rigorously, with appropriate controls, replication, and sample sizes. The conclusions must be drawn appropriately based on the data presented.**

**Reviewer #2: Yes**

**Reviewer #3: Partly**

**3. Has the statistical analysis been performed appropriately and rigorously?**

**Reviewer #2: Yes**

**Reviewer #3: Yes**

**4. Have the authors made all data underlying the findings in their manuscript fully available?

The** [**PLOS Data policy**](http://www.plosone.org/static/policies.action#sharing) **requires authors to make all data underlying the findings described in their manuscript fully available without restriction, with rare exception (please refer to the Data Availability Statement in the manuscript PDF file). The data should be provided as part of the manuscript or its supporting information, or deposited to a public repository. For example, in addition to summary statistics, the data points behind means, medians and variance measures should be available. If there are restrictions on publicly sharing data—e.g. participant privacy or use of data from a third party—those must be specified.**

**Reviewer #2: Yes**

**Reviewer #3: Yes**

**5. Is the manuscript presented in an intelligible fashion and written in standard English?

PLOS ONE does not copyedit accepted manuscripts, so the language in submitted articles must be clear, correct, and unambiguous. Any typographical or grammatical errors should be corrected at revision, so please note any specific errors here.**

**Reviewer #2: Yes**

**Reviewer #3: Yes**

**6. Review Comments to the Author

Please use the space provided to explain your answers to the questions above. You may also include additional comments for the author, including concerns about dual publication, research ethics, or publication ethics. (Please upload your review as an attachment if it exceeds 20,000 characters)**

**Reviewer #2: Thank you for addressing all my previous comments. I really enjoyed reading the revised version and also the sections added based on other reviewers' comments.

Some last suggestions (that you may or may not want to take into account):
1. Discussing the absence of correlations with morphological and phonological awareness at the very end of the discussion might not be the best approach since it is nicer if you conclude with the important findings of your paper.**

RESPONSE: We agree, and have now moved the sections about phonological and morphological awareness on p.35-37 before that about visual training as a potential intervention strategy.

**2. I think the tests you did to check overfitting are informative so you might want to provide this information at some point.**

RESPONSE: We have now added the results of the check for data overfitting at the end of the result section on p.23.

**3. I believe you mention COREVA but then change to COVERA. This is not very important. I felt that this section wasn't very clearly linked to your work. This may be just my feeling but I mentioned the battery because it might be of interest in relation to your work rather than because it had to be mentioned in detail.**

RESPONSE: The typo has now been corrected, and we now clarified, on p.39, that the paragraph on COREVA is to discuss the shared task demand in visual perceptual training and some existing intervention strategies.

**Reviewer #3: I want to reiterate my comments from the previous review that I think that the paper is quite well written and that the analyses appear to be appropriate. I do not have particular problems with this paper being accepted, but I add some comments below for further guidance. I stand by my previous comment that alternative explanations should be discussed further, although the authors have added some discussion on this which is good. It is in the end up to the editor to decide whether further action should be taken to address my comments below, and I do not believe that I need to re-review the paper unless the editor thinks that this is necessary of course.

“…perceptual fluency for characters predicted speeded and non-speeded word reading performance.” I know that the authors do not agree but many would simply say that this means that reading predicts reading.**

RESPONSE: As responded above, we now elaborated in detail why we respectfully disagree that our findings simply showed ‘reading predicts reading’, and why the perceptual fluency measure can emphasize one’s ability in visual discrimination given the possible involvement of subsequent linguistic and memory processes in task performance. This discussion is now on p.29.

**p. 8: “…word reading explicitly requires one to read aloud the words” – most often, people read silently so I do not think you can make such a claim.**

RESPONSE: We have now clarified that our discussion refers to the word reading *tasks* that require one to read words aloud. Silent reading is more likely adopted by proficient readers (Seidenberg, 2005), and is not common in the literature of dyslexia that typically involve beginning readers and requires an explicit measure of word reading performance.

**p. 9: “With a brief time gap between stimulus presentation and report, the requirement on visual working memory is relatively minimal.” I disagree. In your task, the delay period is 500 ms and masked. This task therefore is in essence a visual working memory task almost by definition. Iconic memory shouldn’t last this long, especially with a mask, see e.g.** [**https://www.frontiersin.org/articles/10.3389/fpsyg.2014.00971/full**](https://www.frontiersin.org/articles/10.3389/fpsyg.2014.00971/full)

RESPONSE: We agree that there is a visual working memory component used in the perceptual fluency paradigm because a 500ms blank time is involved. The key here is to what degree the perceptual fluency measure is limited by working memory during this delay period, which should be minimal given the brief time gap. In other words, if they perceive the stimuli well, it is unlikely that they would fail to respond correctly simply because of decay during the working memory delay.

The sentence is now clarified on p.9, 1^st^ paragraph as below:

*‘with a brief time gap between stimulus presentation and report, task performance is likely limited by visual perception instead of visual working memory’*

**p. 9: “A separate perceptual fluency task for four-digit strings was also included. This task, together with RAN, ensured that any explanatory power of the perceptual fluency for characters on Chinese reading would not be explained by visuospatial abilities or discrimination abilities general to all kinds of visual stimuli and objects.” Here, again, I disagree. You yourselves say that “Chinese characters are visually complex” and state that this is one reason why perceptual expertise with words might be particularly important in Chinese reading. Digits are much less visually complex than Chinese characters so the explanatory power of the perceptual fluency for characters for Chinese reading could possibly be explained by e.g. discrimination abilities general to all kinds of visual stimuli and objects.**

RESPONSE: The difficulty of visual shape discrimination, the key ability underlying perceptual expertise, is not simply driven by the complexity of the stimuli, but more critically by the similarity and diagnosticity between the stimuli and its alternatives. For example, learning to recognize the same set of stimuli can be easier when the task involves categorization (objects with components of different basic shapes) than that involves individuation (objects with same basic shapes but subtle differences in the details of the shapes; Wong et al., 2009).

As discussed on p.28, paragraph 2-3, the perceptual fluency measure captures both domain-general abilities (common to recognizing all objects in general) and domain-specific abilities (specific to recognizing characters or to recognizing digits respectively). In this case, since domain-general abilities is common to all objects, it must have been captured and partialed out by percpetual fluency with digits by definition. Domain-specific abilities, driven by the different degrees of similarity or diagnosticity of the objects within a category, is the part that cannot be partialed out by RAN or by the perceptual fluency measures with digits.

**P. 26: „In the current study, the tasks... would have been too easy and resulted in ceiling effects with typical readers.“ You cannot say this as some of the tasks were staircase tasks and therefore have no ceiling/floor effects by definition.**

RESPONSE: We have now clarified on p.26 that only ‘some of the tasks’ would result in the ceiling effects.

**P. 29: „Since domain-general object recognition skills should be captured by both perceptual fluency measures, we did not observe any evidence for the role of domain-general skills in predicting word reading.“ This cannot be be claimed, as perceptual fluency for digits (tapping into domain-general skills, among other things) DID predict word reading, i.e. it was correlated with it. However, the character fluency task (presumably tapping into domain-specific skills) explained further variance not explained by the digit task.**

RESPONSE: This sentence is now clarified as following on p. 29:

*we did not observe any evidence for the role of domain-general skills in uniquely predicting word reading in multiple regression analyses.*

**P. 30: „...perceptual fluency task with digits were identical to that with characters in terms of how the stimuli were spatially presented, and therefore general visual crowding effects should have been partialed out in our regression model by the perceptual fluency task with digits.“ I would be careful in making such as claim, as characters are likely more self-crowding and more visually complex than numbers, as pointed out in my previous review.**

RESPONSE: On p.33, we have now clarified that the discussed visual crowding effects referred to that constrained by eccentricity (Bouma, 1970). At the end of that paragraph, we also clarified that there could be additional factors affecting the degree of visual crowding experienced by each specific stimulus category. The sentences are also included below:

*Note that the visual crowding discussed here largely concerns the between-object visual crowding, that is largely constrained by eccentricity (85,118,119,121). Additional factors may affect the exact degree of visual crowding experienced with a specific stimulus category, such as target complexity, flanker complexity, the similarity and complexity differences between target and flankers, and self-crowding (122–124). How these factors affect the category-specific visual crowding experienced during the perceptual fluency measure with characters and digits is unclear, and further studies should examine this and clarify its relationship with perceptual expertise.*

**Minor details: „Perceptual expertise with words might be particularly important in Chinese reading because of two reasons“ (p. 5) should read: „Perceptual expertise with words might be particularly important in Chinese reading because of three reasons“. „**

RESPONSE: This is now corrected.

**Recent studies have reported impaired performance in object recognition in children with developmental dyslexia“ (p. 28) should read: „Recent studies have reported impaired performance in object recognition in adults with developmental dyslexia“.**

RESPONSE: This is now corrected.

**7. PLOS authors have the option to publish the peer review history of their article (**[**what does this mean?**](https://journals.plos.org/plosone/s/editorial-and-peer-review-process#loc-peer-review-history)**). If published, this will include your full peer review and any attached files.


If you choose “no”, your identity will remain anonymous but your review may still be made public.


Do you want your identity to be public for this peer review? For information about this choice, including consent withdrawal, please see our** [**Privacy Policy**](https://www.plos.org/privacy-policy)**.**

**Reviewer #2: No**

**Reviewer #3: No**

**[NOTE: If reviewer comments were submitted as an attachment file, they will be attached to this email and accessible via the submission site. Please log into your account, locate the manuscript record, and check for the action link "View Attachments". If this link does not appear, there are no attachment files.]

While revising your submission, please upload your figure files to the Preflight Analysis and Conversion Engine (PACE) digital diagnostic tool,** [**https://pacev2.apexcovantage.com/**](https://pacev2.apexcovantage.com/)**. PACE helps ensure that figures meet PLOS requirements. To use PACE, you must first register as a user. Registration is free. Then, login and navigate to the UPLOAD tab, where you will find detailed instructions on how to use the tool. If you encounter any issues or have any questions when using PACE, please email PLOS at** [**figures@plos.org**](mailto:figures@plos.org)**. Please note that Supporting Information files do not need this step.**
